# The Bacterial Volatile Organic Compound *N,N*-Dimethylhexadecylamine Induces Long-Lasting Developmental and Immune Responses throughout the Life Cycle of *Arabidopsis thaliana*

**DOI:** 10.3390/plants12071540

**Published:** 2023-04-03

**Authors:** Christian Hernández-Soberano, José López-Bucio, Eduardo Valencia-Cantero

**Affiliations:** Instituto de Investigaciones Químico Biológicas, Universidad Michoacana de San Nicolás de Hidalgo, Morelia 58030, Michoacán, Mexico; christian.hernandez@umich.mx

**Keywords:** long-lasting responses, jasmonic acid pathway, ethylene pathway, necrotrophic pathogen, pathway crosstalk

## Abstract

*N,N*-dimethylhexadecylamine (DMHDA) is a bacterial volatile organic compound that affects plant growth and morphogenesis and is considered a cross-kingdom signal molecule. Its bioactivity involves crosstalk with the cytokinin and jasmonic acid (JA) pathways to control stem cell niches and induce iron deficiency adaptation and plant defense. In this study, through genetic analysis, we show that the DMHDA-JA-Ethylene (ET) relations determine the magnitude of the defensive response mounted during the infestation of *Arabidopsis* plants by the pathogenic fungus *Botrytis cinerea*. The *Arabidopsis* mutants defective in the JA receptor *CORONATINE INSENSITIVE 1* (*coi1-1*) showed a more severe infestation when compared to wild-type plants (Col-0) that were partially restored by DMHDA supplements. Moreover, the oversensitivity manifested by *ETHYLENE INSENSITIVE 2* (*ein2*) by *B. cinerea* infestation could not be reverted by the volatile, suggesting a role for this gene in DMHDA reinforcement of immunity. Growth of Col-0 plants was inhibited by DMHDA, but *ein2* did not. Noteworthy, *Arabidopsis* seeds treated with DMHDA produced more vigorous plants throughout their life cycle. These data are supportive of a scenario where plant perception of a bacterial volatile influences the resistance to a fungal phytopathogen while modulating plant growth.

## 1. Introduction

Plants co-evolved with microorganisms during their diversification and colonization of the terrestrial environment, and appropriate crosstalk enabled mutual benefits throughout their life cycles [1]. Most microbes, including bacteria and fungi, inhabit a discrete soil patch termed the rhizosphere, where itsphysical and chemical properties change due to the chemotactic and nutritional effects of root exudates [2]. Taken as a whole, the genome of the rhizospheric microbiome is large enough so that plants receive many benefits from microbial functions, which leads to the concept that microbe genomes represent a second plant genome [3,4], providing plants with adaptive traits related to nutrient acquisition and tolerance to biotic and abiotic stress, something comparable to the gut microbiome, which benefits mammal hosts [5]. In this sense, a healthy microbiome contributes to plant growth and survival, whereas alterations in its composition lead to plant dysbiosis, stress and disease [6,7].

Plants are frequently exposed to phytopathogens and have evolved mechanisms to fight them [2]. These mechanisms operate through the crosstalk of the canonical phytohormones jasmonic acid (JA), ethylene (ET), and salicylic acid (SA), which acting through well-defined signaling pathways, act as the backbone of the plant immune system [8,9]. This allows the recognition of structural molecules from pathogens, diffusible molecules that act as elicitors, or volatiles that upon recognition, enable plants to mount a strong and effective resistance [10].

Plant defense responses are determined mainly by the pathogen attack strategies; as such, necrotrophic pathogens that usually damage the host tissues activate the JA/ET pathway, which in turn leads to the production of defensins [11,12]. Species of beneficial bacteria as part of a healthy microbiome also induce the JA/ET pathway; these bacteria do not really attack their host but produce a diversity of compounds that elicit the plant defense responses [13,14]. Boosting immunity is costly, as it consumes energy in detriment for plant growth [15]. Application of some metabolites such as β and γ-aminobutyric acids, BABA and GABA, respectively, or the tricarboxylates citrate and fumarate, induces a priming state that strengthens the plant immune system, that is, induces priming at a low energetic cost and considerably reduce damage upon pathogen challenge [16,17,18]. Beneficial bacteria, as well as their compounds, also prime the plant immune system [14,19]. Among the bacterial compounds, *N*-acyl-*L*-homoserine lactones (AHLs) are particularly interesting, acting as highly specific communication systems among bacterial species, also termed quorum-sensing systems. Detection of AHLs has been reported in plants; small AHLs ranging from four to eight carbons in length promote defense priming, whereas longer acyl-chained compounds restrict root growth and induce the formation of lateral roots and root hairs [20,21,22]. Another example of this cross-kingdom signaling involves the volatile organic compounds (VOCs) emitted by rhizospheric bacteria, some of which modulate the hormonal status of the plant while improving the capacity of roots to access unavailable pools of mineral nutrients [23,24].

A pioneering work by Ryu et al. (2003) [25] demonstrated that strains of *Bacillus amyloliquefaciens* promote *Arabidopsis* growth through the emission of the VOCs acetoin and their metabolic precursor 2,3 butanediol. Since then, a wide diversity of plant growth-promoting rhizobacteria have been reported to produce different specific effects on plants by means of VOCs emission [26,27]. Mechanisms involved in these effects include the modulation of endogenous phytohormone levels [28], access to soil nutrients [29], and protection against necrotrophic pathogens such as *Botrytis cinerea* [30]. *B. cinerea* is extensively employed as a model in studies of plant defense responses due to its wide host spectrum, its rich multilayer pathogenic toolbox, and its economic relevance [31,32].

*N*,*N*-dimethylhexadecylamine (DMHDA) is a VOC produced by diverse plant-beneficial bacteria, such as *Pseudomonas fluorescens*, *Bacillus* sp., and *Arthrobacter* sp. that belong to the major phyla proteobacteria, firmicutes, and actinobacteria, respectively [33,34,35]. DMHDA acts as a signal molecule for bacteria–bacteria communication and has the potential to modulate the endophytic plant microbiome [36,37,38]. In a comparable manner to AHLs, DMHDA could induce defense responses and promote plant growth through different mechanisms [39,40]. Plant perception of DMHDA involves the cytokinin receptor AHK2 and implies the crosstalk of cytokinin and JA pathways at different levels. It induces JA-dependent gene expression that is antagonized by cytokinins, thus modulating cell proliferation and identity of stem cell niches [41,42,43]. However, it is at present unknown how the endogenous hormonal balance influenced by DMHDA affects plant behavior under pathogen challenge.

## 2. Results

### 2.1. DMHDA Represses Growth of Arabidopsis Plants In Vitro

Previously, we reported that DMHDA induces resistance in strawberry plants against *B. cinerea* at the expense of growth [40]. However, the regulatory mechanisms implicated in this phenomenon remained unknown. In the present work, we used the model plant *A. thaliana* to understand the genetic bases of the growth/defense tradeoff analysis.

**Figure 1:**
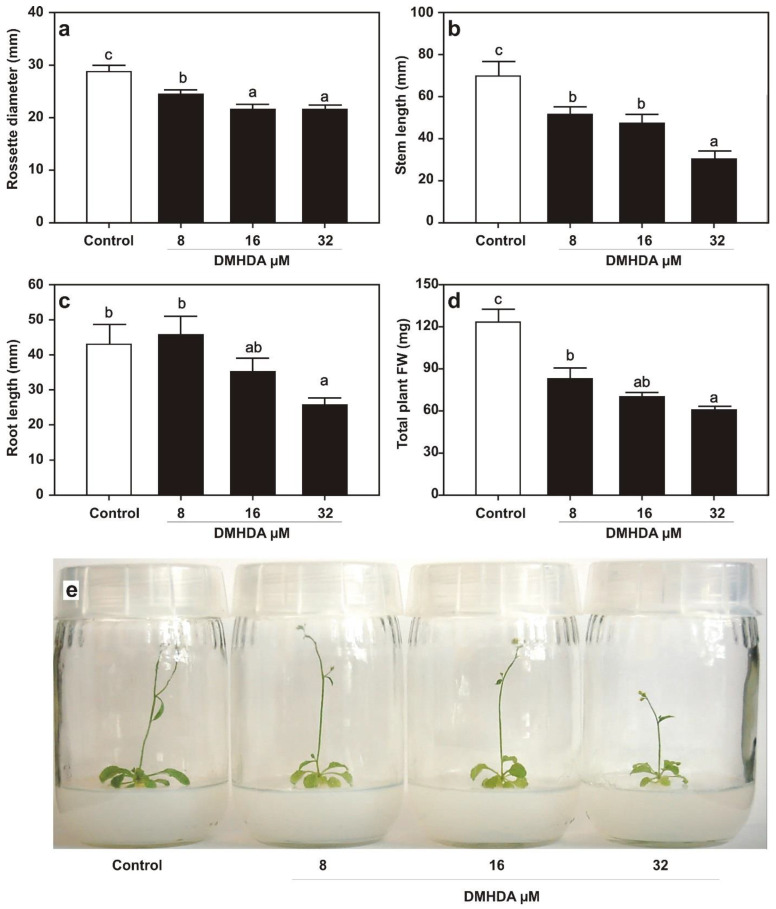
**Effect of DMHDA on *A. thaliana* growth.** Ten days after germination on Petri plates with 0.2× MS Medium, seedlings were transferred to flasks with 0.2× MS medium supplied with 0, 8, 16, and 32 µM of DMHDA; eight days after transfer, phenotypical parameters were recorded: (**a**) Rosette diameter, (**b**) stem length, (**c**) root length, and (**d**) total plant fresh weight (FW). Panel (**e**) shows representative photographs of plants. Values represent the means of ten plants (one plant per flask) ± SE. Different letters represent statistically different means (Duncan’s test; *p* < 0.05).

First, the effect of DMHDA on *A. thaliana* growth was analyzed by supplementing 8, 16, and 32 µM concentrations of the compound directly to the culture medium. DMHDA reduced the growth of *A. thaliana* in most of the concentrations applied with significantly lower rosette diameter (Figure 1a), stem length (Figure 1b), root length (Figure 1c), and plant weight (Figure 1d) than those grown in the control condition. Figure 1e shows the phenotypes of plants grown into the flasks with 0.2× MS medium supplied with the above-mentioned DMHDA concentrations and the growth-repressing effects of the compound.

### 2.2. DMHDA Protects Arabidopsis Leaves from B. cinerea Infection

**Figure 2:**
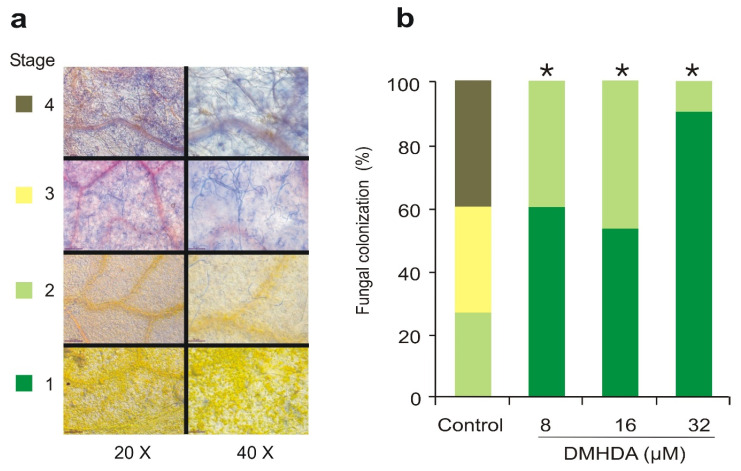
**DMHDA promotes the resistance of *A. thaliana* seedlings against *Botrytis cinerea*.** Leaves of *A. thaliana* plants were infected with *B. cinerea* and, five days after inoculation, were stained with trypan blue. Panel (**a**) shows the severity of symptoms considered to classify the colonization by *B. cinerea* in leaves of *A. thaliana*. Representative examples of infection classes used for quantification of *B. cinerea* infection. Stage 1, absent or minimal colonization; stage 2, ≤50% leaf area colonized by the pathogen; stage 3, ≤75% leaf area colonized by the pathogen, presence of conidiophores; stage 4, >75% leaf area colonized by the pathogen, and production of abundant conidiophores. Representative photographs from a Leica DFC450 C microscope at 20× and 40× objectives. Panel (**b**) shows fungal colonization of plants grown in 0.2× MS medium supplied with 0, 8, 16, and 32 µM DMHDA and infected with *B. cinerea*. Three days after inoculation, 50 leaves per treatment were stained with trypan blue and classified according to the severity symptoms shown in Figure 1a. Asterisks (*) indicate statistically significant differences in stages distribution compared to the control (0 µM DMHDA) condition (χ^2^ test; *p* < 0.05).

Next, the effect of DMHDA on the resistance of *A. thaliana* against *B. cinerea* infection was tested. For this purpose, four stages of severity-increasing fungal infection were considered, where stage 1 describes absent or minimal fungal colonization, and stage 4 indicates abundant fungal colonization with the production of conidiophores over plant tissues (Figure 2a). Five days after inoculation with *B. cinerea*, all the analyzed leaves from the plants grown in control conditions showed some degree of infection, ranging from 2 to 4 infection stages (approximately a third of the leaves in each stage). In contrast, plants treated with DMHDA developed a notorious and statistically significant resistance against *B. cinerea* infection since most of the analyzed leaves were located at stage 1 of the infection (absent or minimal colonization) and the rest at stage 2. Among these, the higher resistance was found in plants treated with 32 µM DMHDA, where only 7.5% of the leaves were colonized (Figure 2b).

### 2.3. Contrasting Phenotypes in The Loss-of-Function of COI1 and EIN2 Genes for the Growth Response to DMHDA

**Figure 3:**
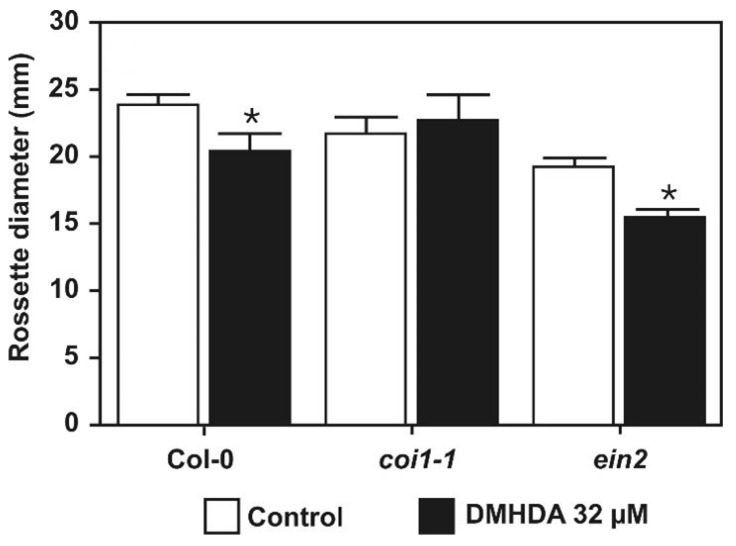
**Role of *COI1* and *EIN2* genetic elements in the growth of *A. thaliana* in response to DMHDA.** Ten days after germination on the Petri dish, seedlings of Col-0, *coi1-1*, and *ein2* were transferred to flasks with 0.2× MS medium supplied with 0 (solvent; Control) and 32 µM of DMHDA. Eight days after transfer, rosette diameter was recorded. Values represent the means of 40 ± SE. Asterisks (*) represent statistically significant differences in rosette diameter compared with the control condition (Student *t*-test; *p* < 0.05).

In order to examine the function of critical elements in the defense phytohormone network, the growth response to DMHDA was compared for the WT (Col-0) and *coronatine insensitive 1* (*coi1*) mutant defective on the jasmonic acid receptor [44], and *ethylene insensitive 2* (*ein2*) altered in a critical component of ethylene sensing [45,46]. Plants were cultivated in medium with the solvent only or supplemented with 32 µM DMHDA; as expected, WT (Col-0) plants had significantly lower rosette diameter under DMHDA than solvent-treated plants. Interestingly, *coi1-1* and *ein2* mutant lines had contrasting phenotypes, being insensitive or sensitive, respectively, to the repressing effects of the volatile regarding rosette diameter (Figure 3). These results uncover novel and opposite roles for jasmonic acid/ethylene signaling in the growth response of plants to a bacterial volatile.

### 2.4. EIN2 Plays an Important Role in DMHDA-Elicited Plant Resistance to B. cinerea 

**Figure 4:**
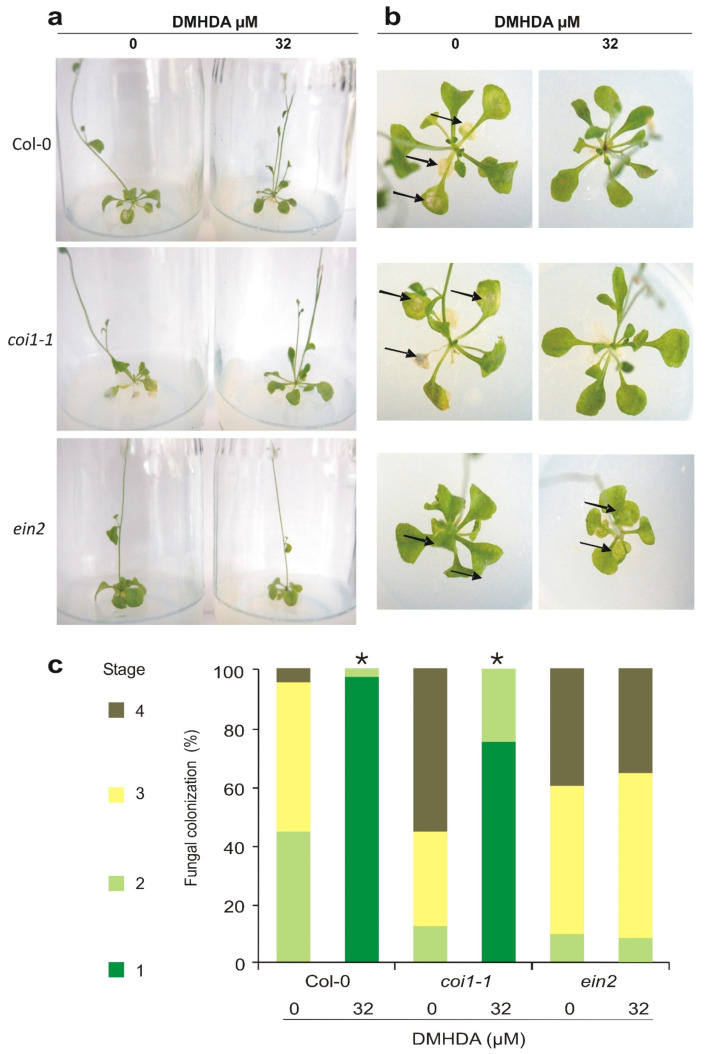
**Role of jasmonic acid and ethylene pathways in the *A. thaliana* resistance against *B. cinerea*.** Arabidopsis Col-0, *coi1-1* and *ein2* seedlings were cultivated in 0.2× MS medium supplied with 0 (solvent) and 32 µM of DMHDA. Plants were inoculated with 1 × 10^5^ conidia/leaf of *B. cinerea*. Panel (**a**) shows representative photographs of a lateral view of plants three days after inoculation. Panel (**b**) view of plant rosette three days after fungal inoculation, black arrows indicate the damage caused by fungus *B. cinerea*. Panel (**c**) quantitation of fungal infection level in *A. thaliana* Col-0, *coi1-1*, and *ein2*. 50 leaves of each plant genotype and treatment were stained with trypan blue and classified. Asterisks (*) indicate statistically significant differences in the infection stages distribution of each plant genotype (independently) compared to the control (solvent only) condition (χ^2^ test; *p* < 0.05).

In plants, the jasmonic acid and ethylene pathways converge to strengthen the immune response against necrotrophic pathogens such as *B. cinerea* [47]. Taking into account the reference scale of symptoms shown in Figure 2b, untreated Col-0 plants showed 32%, 36%, and 32% in stages 2, 3, and 4, clearly manifesting the progress of the infection. In contrast, DMHDA induced the resistance of Col-0 plants against the pathogen (Figure 4a,b), with 80% and 20% in stages 1 and 2 of infection, respectively. Solvent-treated *coi1-1* plants showed higher susceptibility to infection by *B. cinerea*. However, the treatment with DMHDA reduced the fungal colonization, showing infestation comparable to Col-0 DMHDA-treated plants, 75% and 25% at levels 1 and 2 of infection severity, respectively (Figure 4c). Solvent-treated *ein2* mutants also showed high susceptibility to fungal colonization, even more so than *coi1-1*, and the DMHDA treatment did not protect them from the fungal spread (Figure 4). These results showed the critical role of EIN2 as a mediator in the plant immunity induced by DMHDA.

### 2.5. Temporal Application of DMHDA Promotes Growth of A. thaliana

**Figure 5:**
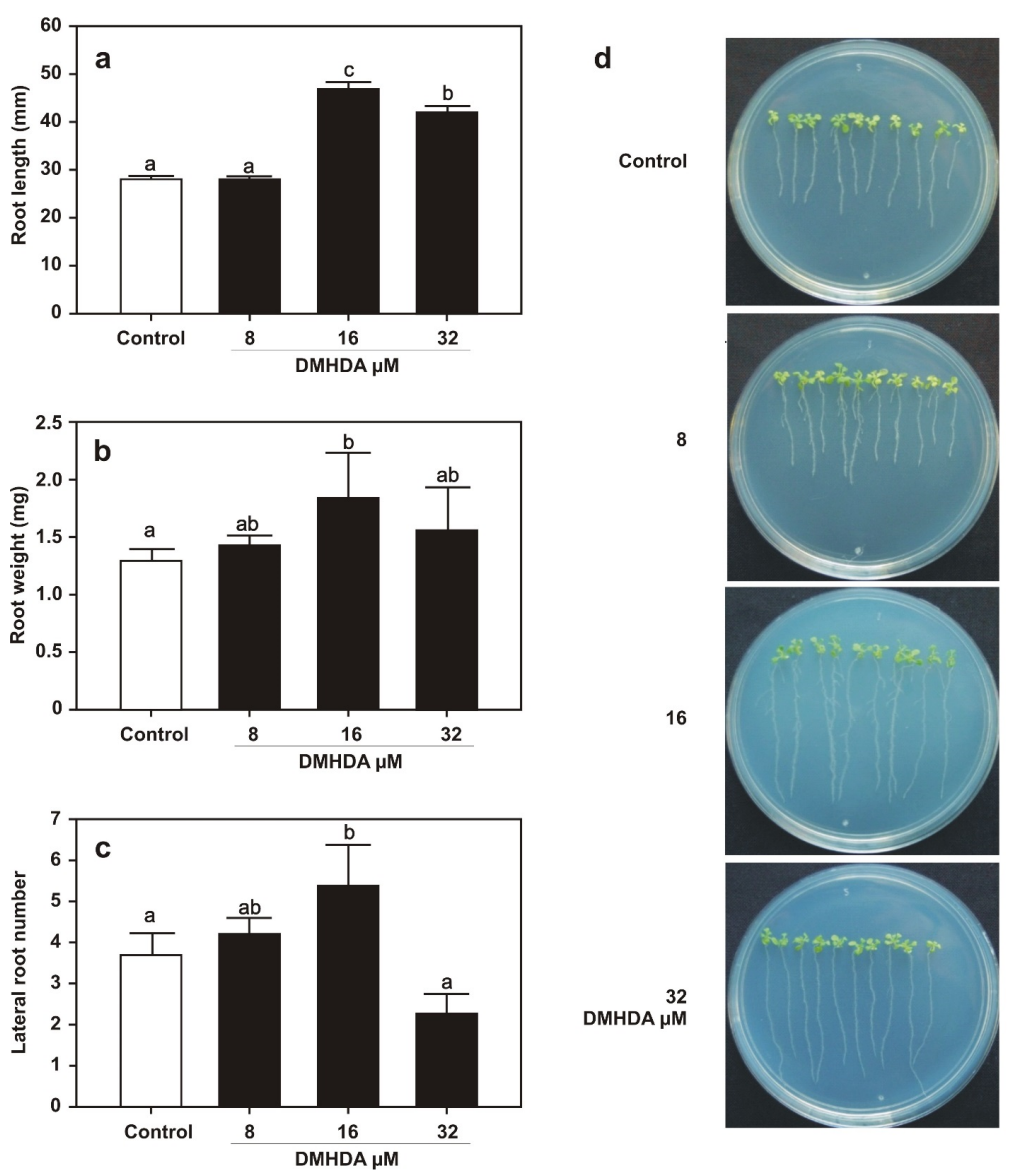
**DMHDA induces long-lasting development effects in vitro in *A. thaliana*.** *A. thaliana* seeds were sown in 0.2× MS medium supplied with 0, 8, 16, and 32 µM of DMHDA. After 72 h after germination, seedlings were transferred to 0.2× MS DMHDA-free plates; after eight days, the morphometrical parameters were recorded: (**a**) root length, (**b**) root weight, and (**c**) lateral root number. Values represent the means of 40 replicates ± SE. Different letters represent means that are statistically different (Duncan´s test; *p* < 0.05). Panel (**d**) shows representative images of the experiment on DMHDA-free plates.

**Figure 6:**
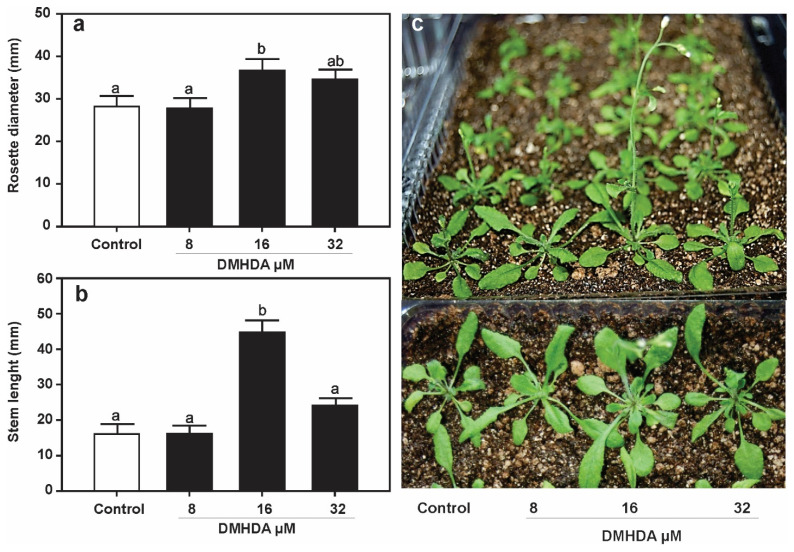
**DMHDA generates long-lasting effects throughout the life cycle of *A. thaliana*.** *A. thaliana* seedlings were germinated for 72 h with DMHDA (0, 8, 16, 32 µM), and then transferred to 0.2× MS DMHDA-free plates; after ten days, plants were transplanted to substrate mixture to complete their life cycle, after three weeks rosette diameter (**a**) and stem length (**b**) were recorded, and representative photographs (**c**) were obtained. Values represent the means of 20 replicates ± SE. Different letters represent means that are statistically different (Duncan´s test; *p* < 0.05).

The continuous application of DMHDA favored defense and compromised plant growth. In order to understand whether temporal application of the volatile could also lead to growth repression or not, *Arabidopsis* WT seedlings were germinated in media with DMHDA for 72 h and then transferred to Petri dishes with fresh MS 0.2× medium without the compound. Ten days after transfer, several plant traits were analyzed, including root length, root weight, and lateral root formation. Plants that were germinated on media with 16 and 32 µM DMHDA produced primary roots significantly longer than those germinated without the volatile, while higher root weight and lateral root number were observed at the lower dose (Figure 5a–c). Representative images of the growth of plants in the Petri plates are presented in Figure 5d. At this stage, plants were transferred from the Petri dishes to plastic containers with a soil substrate mixture and cultured during their entire life cycle. At the reproductive stage, rosette diameter and stem length were measured, and representative photos were taken from the plants grown in the soil. The plants temporally exposed to 16 µM DMHDA were greater than control plants, with a 1.25-fold increase of rosette diameter and a 2.6-fold higher stem length, whereas plants exposed to 8 or 32 µM DMHDA prior to transfer to soil did not show significant differences on growth compared with controls (Figure 6a–c). These results show the dynamic growth responses of *Arabidopsis* to DMHDA that are influenced by the concentration of the compound, the duration of treatment, and the plant trait.

### 2.6. DMHDA Induces Long-Lasting Effects on The Immune Response of Arabidopsis Plants

**Figure 7:**
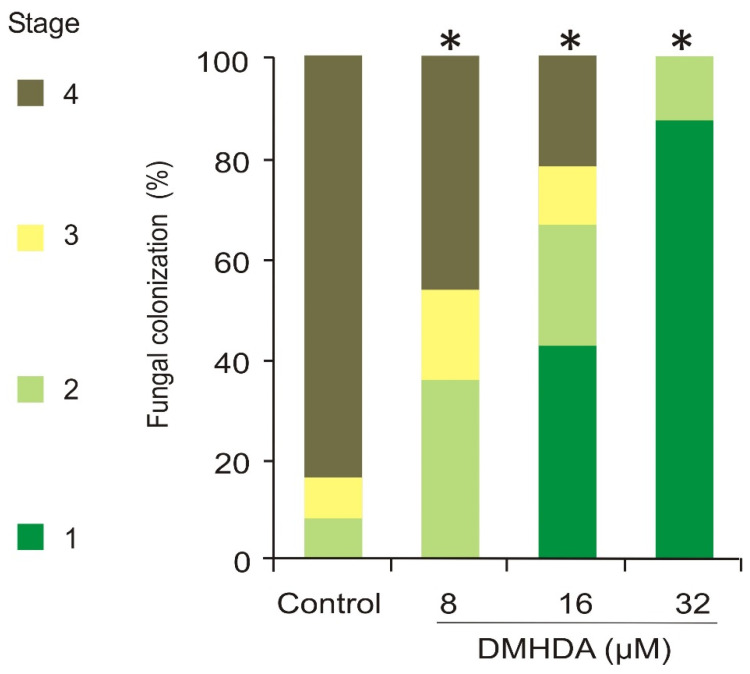
**DMHDA induces a long-lasting effect on the immune response of *A. thaliana*.** *A. thaliana* seedlings were germinated for 72 h with DMHDA (0, 8, 16, 32 µM) and then transferred to 0.2× MS DMHDA-free plates; after ten days more, plants were transplanted to substrate mixture and cultured for three weeks before inoculation with 1 × 10^5^ conidia/leaf of *B. cinerea*. Three days after inoculation, 100 leaves of each treatment were stained with trypan blue and classified. Asterisks (*) indicate statistically significant differences in infection stages distribution for each plant treatment (independently) compared to the control (solvent only) condition (χ^2^ test; *p* < 0.05).

Plants germinated on DMHDA and grown in a soil substrate mixture for three weeks were inoculated with *B. cinerea*, and three days after infection, the symptoms were evaluated. The resistance of plants to the pathogen was related to the concentration of DMHDA where plants were germinated (Figure 7). All the leaves in control plants were infected, 84% in the infection stage 4, and among the remaining leaves, 8% were in stage 3 and 8% in stage 2. However, all plants germinated on DMHDA showed significant differences from the control plants; plants germinated in 8 µM DMHDA showed 46% in stage 4 of infection, 18% in stage 3 and 36% in stage 2; for plants germinated in 16 µM DMHDA, only 21% were in stage 4 of infection, while leaves in stage 1 were 43%. Notoriously, among plants germinated on 32 µM DMHDA, 12% of leaves were in stage 2, and the remaining 88% were in stage 1, which denotes pathogen absence or minimal colonization (Figure 7). These results show that the DMHDA induces a priming effect against the necrotrophic pathogen *B. cinerea* during germination that protects the plants from an infection produced later on in development.

## 3. Discussion

Plants establish perdurable relationships with their microbiomes through sensing and response to a wide range of bacterial metabolites, including diffusible molecules and volatile organic compounds. The autoinducer molecules, also termed quorum-sensing compounds, include AHLs, for which dual roles in growth and defense responses have been established [48,49]. The fact that the growth/defense tradeoff relies on the size of the molecule indicates that defense priming and plant growth promotion may transit in different ways. Previously, we showed that the bacterial compound DMHDA produced a priming defense effect and modulated plant growth in strawberry plants [40]; in the present work, we explored the signaling pathways stimulated by DMHDA and the long-lasting effects produced by this compound using the model plant *Arabidopsis*.

The effect of DMHDA was assessed at several low micromolar concentrations, whose continuous exposure had inhibitory effects on plant growth. Our data are consistent with those of Vázquez-Chimalhua et al. [42,43] in that 32 µM strongly inhibited root growth. Noteworthy, plants grown with DMHDA showed a very notorious resistance against infection with the necrotic pathogen *B. cinerea*, and these data are in agreement with previous research in *Fragaria* × *ananassa* plants [40]; thus, we conclude that its bioactivity is of broad applicability to not closely related plant species. DMHDA itself has a moderate inhibitory effect on *B. cinerea* when the mycelium is grown in culture media with the compound [50]. In the present work, *B. cinerea* was inoculated on the *Arabidopsis* leaves, and direct contact between the fungus and DMHDA was avoided; however, a slight inhibitory effect of DMHDA on *B. cinerea* was considered possible. In this sense, we employed mutant plants compromised in two major defense pathways to dissect the plant immunity participation in the DMHDA effect against the fungus.

It is well established that a combination of ET and JA accumulation promotes defense against necrotrophic pathogens [51], and indeed DMHDA cross-talks with the JA pathway [39,52]. Other compounds, such as the polyamines spermine and spermidine, and fructans from different sources, including microorganisms prime reactive oxygen species (ROS) dynamics, and confer resistance to *Arabidopsis* plants against *B. cinerea* [53,54]. The possible participation of JA and ET pathways in the DMHDA effect on *Arabidopsis* plants was investigated using selected mutants in master genes of these pathways *(coi1-1* and *ein2* mutants). While the growth of *coi1-1* did not apparently differ from the WT in medium supplemented with DMHDA, *ein2* plants showed growth inhibition. This indicated that the protein product encoded by *EIN2*, possibly as part of the ET signaling pathway, is not involved in growth modulation, but the JA pathway is. Previous findings showed that DMHDA modulates *Arabidopsis* root growth by modifying the balance between stem cell niche and JA-dependent gene expression [43]; the current results point to the fact that DMHDA does not replace the JA pathway but requires the JA COI1 receptor to modulate plant growth. On the other hand, *coi1-1* plants grown in DMHDA medium showed a clear priming effect against *B. cinerea* infection comparable to that of wild-type mutants, suggesting a scenario where DMHDA induces defense priming independently of COI1, and in this sense, does not act as an analogous of JA, suggesting a downstream role. On the contrary, DMHDA did not rescue the wild-type phenotype in *ein2* mutants, showing that defense priming by DMHDA needs an intact ET pathway to be effective and suggesting that the confluence of DMHDA/JA pathway with ET pathway is required for this effect.

The siblings of plants from DMHDA-treated seeds were resistant to the pathogen showing a priming long-lasting effect. In works using BABA as a priming agent, the priming was associated with PTI [55] and required the regulatory protein NPR1 that controls SA-signaling for the modulation of the methylome [56,57]. From our research, the crosstalk DMHDA-JA suggests an alternative for the priming effect of the volatile since JA itself is a priming agent against necrotrophic pathogens [58], and consistently enhanced JA levels in *Arabidopsis* led defense priming to pass to the offspring [59]. 

Metabolites, such as BABA and GABA, are naturally present in plants, enhancing their concentrations in stress conditions and inducing a priming effect on plant defense at the time they compromise plant growth [60,61,62]. In summary, we have shown the long-lasting effects of the bacterial VOC DMHDA on plant growth and defense priming and demonstrated that the growth/defense tradeoff is dynamically modulated by JA/ET-signaling, which broadens the spectrum of bacterial metabolites to make plants more resilient.

## 4. Materials and Methods

### 4.1. Plant Material and Growth Conditions

Arabidopsis (*Arabidopsis thaliana*) ecotype Col-0, *coi1-1* [63], and *ein2* [45] mutants were used for the experiments. Seeds were disinfected with 95% (*v*/*v*) ethanol for 5 min and 20% (*v*/*v*) bleach for 7 min. After careful washing with sterilized deionized water, seeds were germinated and grown on agar plates containing 0.2× Murashige and Skoog medium (Murashige and Skoog basal salts mixture, M5524; Sigma; St Louis MO, USA), pH 7, 0.6% (*w*/*v*) sucrose, and 1% (*w*/*v*) agar plant TC (micropropagation grade, A111; Phytotechnology Laboratories). Plates were placed vertically at an angle of 65° to allow root growth along the agar surface and unimpeded hypocotyl growth into the air. For plant growth, we used a plant growth chamber (Percival Scientific Inc., AR66L) with a photoperiod of 16 h of light, 8 h of darkness, a light intensity of 100 µmol m^−2^/s^−1^, and a temperature of 22 °C (standard conditions).

### 4.2. Propagation of Botrytis cinerea

The phytopathogenic necrotrophic fungus *B. cinerea* BC2 [50] was used in this study. The strain identification was confirmed by PCR amplification of a 159-bp segment of the *B. cinerea* specific marker sequence C729+/C29− [64] using oligonucleotides BCN1F (5′ CCT GGG TTG TTG CTA TCC TTT ATC 3′) and BCN1R (5′ GGC GTC GTT GGT GAG TGG 3′) [40]. *B. cinerea* BC2 was routinely maintained in potato dextrose agar (211,900; BD Bioxon,) plates and incubated at 25 °C in darkness until sporulation. Conidia were collected by adding deionized sterile water to the Petri dishes, and then the suspension was collected with a micropipette. Conidia quantification was performed using a Neubauer hemacytometer (Hausser Scientific, Horsham, PA, USA).

### 4.3. Chemicals

*N,N*-dimethylhexadecylamine (DMHDA) was purchased from Sigma-Aldrich, kept at 4 °C, and dissolved in ethanol prior to its use. For controls, equal volumes of solvents were used as in the highest DMHDA treatment.

### 4.4. Analysis of Plant Growth

The parameters related to plant growth were measured as follows: for primary root and stem length as well as rosette diameter, a digital Vernier caliper (Mitutoyo Corporation, Tokyo, Japan, catalog 500-196-30) was employed; lateral root number (present in the primary root) was counted using a stereoscopic microscope (Leica EZ4D, Leica Microsystems, Wetzlar, Germany); and shoots and roots were weighed in an analytical scale (TE64, Sartorius, Goettingen, Germany).

### 4.5. Quantification of B. cinerea Colonization in Arabidopsis Leaves

The leaves were classified into four infection stages as follows. Five days after *B. cinerea* inoculation, 50 (100 in the case of the substrate experiment) leaves of each treatment were randomly selected, stained with trypan blue as described [65] and classified according to four stages of the colonization scale distinguished as follows: stage 1, absent or minimal colonization; stage 2, ≤50% leaf area colonized by the pathogen; stage 3, ≤75% colonized area, presence of conidiophores; stage 4, >75% leaf area colonized, abundant conidiophores and conidia. Colonization was determined using a stereoscopic microscopic (Leica EZ4D) at a magnification of 20 and 40 X.

### 4.6. Effect of DMHDA on Growth and Immune Response of Arabidopsis

Ten days after germination, *Arabidopsis* Col-0, *coi1-1* (JA receptor mutant), and *ein2* (ethylene signal transducer mutant) plants were transferred to 170 mL glass flasks containing 30 mL MS 0.2× culture medium added with DMHDA to obtain final concentrations of 8, 16, and 32 µM and ethanol for control conditions for eight days. After that time, half of the plants from each treatment were inoculated with 5 µL of 1 × 10^5^ conidia/mL suspension of *B. cinerea* in five leaves at the comparable developmental stage. Three days later, random leaves were collected and stained with trypan blue, mounted in microscope slides, and classified in four infection stages scale according to microscopical observations, and the other half of the plants were measured for growth parameters (rosette diameter, stem length, root length, and total plant fresh weight).

### 4.7. DMHDA Long-Lasting Effect on The Growth of A. thaliana

To test if DMHDA was capable of inducing a long-lasting developmental effect on *A. thaliana*: first, DMHDA was added to a germination medium; after 72 h, once the cotyledons emerged, the plantlets were transferred to Petri dishes with MS 0.2× medium without DMHDA, to ensure that only the 72 h molecule stimuli be responsible for the effect on *A. thaliana* life cycle. Eight days after transplanting, primary root length, lateral root number, shoot, and root weight were registered. To follow the changes throughout the life cycle of *A. thaliana*, plants obtained under the same system described above were transplanted to plastic containers (24 × 15 cm) with 800 g of substrate mixture (60 min autoclave sterilized) composed of organic matter mix 3; perlite and premium vermiculite (Sun Gro Horticulture; Vancouver, Canada) in a 3:1:1 proportion, respectively, and transplanted plants were again placed into a plant growth chamber to analyze and measure changes in development (rosette diameter and stem length) during their life cycle.

### 4.8. DMHDA Long-Lasting Effect on The Immune Response of A. thaliana Plants

For the long-lasting resistance assay, under the same conditions (after 72 h of DMHDA treatment, in germination medium), the plantlets were transferred to Petri dishes with MS 0.2× medium without DMHDA to ensure that only the 72 h molecule stimuli be responsible for the effect on *Arabidopsis* life cycle. Then, plants were transplanted to plastic containers (24 × 15 cm) with a substrate mixture (60 min. autoclave sterilized) and placed in a plant growth chamber under standard growth conditions. Seven days after transfer, the leaves were inoculated with 5 µL of 1 × 10^5^ conidia/mL suspension of *B. cinerea* at the comparable developmental stage. Five days after inoculation, leaves from each treatment were randomly collected, trypan blue stained, and classified into four infection stages scale according to detailed microscopical observations.

### 4.9. Statistical Analysis

Data were analyzed with the ANOVA test and Duncan´s means separation test for multiple comparisons; (*p* ≤ 0.05). For percentage analysis, it was used a proportion analysis followed by the χ^2^ test. To percentage of *B. cinerea* colonization (4 stages scale) χ^2^ test (*p* ≤ 0.05).

## Data Availability

Data will be available upon request to the corresponding authors.
